# A synthetic cell-penetrating peptide derived from nuclear localization signal of EPS8 exerts anticancer activity against acute myeloid leukemia

**DOI:** 10.1186/s13046-018-0682-x

**Published:** 2018-01-22

**Authors:** Yiran Chen, Xiaoling Xie, Anqin Wu, Lei Wang, Yuxing Hu, Honghao Zhang, Yuhua Li

**Affiliations:** Department of Hematology, Zhujiang Hospital, Southern Medical University, No. 253 GongyeDadaoZhong, Guangzhou, Guangdong 510282 People’s Republic of China

**Keywords:** Epidermal growth factor receptor pathway substrate no.8 (EPS8), Acute myeloid leukemia, Peptide, Nuclear localization signal

## Abstract

**Background:**

Oncogenic roles of epidermal growth factor receptor pathway substrate no.8 (EPS8) have been widely reported in various tumors, making targeting of EPS8 an appealing prospect. Here, we describe the role of EPS8 in acute myeloid leukemia (AML) and consider the potential of EPS8 as an anti-AML target. Nuclear localization signal (NLS) residues of tumor-associated proteins are crucial for cell cycle progression, and specific inhibitors derived from the NLS have inhibitory effect on cancer cells. The NLS in EPS8 has potential as a specific anti-AML target.

**Methods:**

Gene Expression Omnibus expression profiles of AML patients were used to test associations between EPS8 expression and AML patient outcome. The biological characteristics of AML cells after EPS8 knockdown were analyzed in vitro and in vivo. A specific peptide (CP-EPS8-NLS) derived from the NLS of EPS8 (amino acids 298–310) was synthesized, and the anti-AML effects of CP-EPS8-NLS were analyzed in cancer cells and in xenograft models. Mutated CP-EPS8-NLS and penetratin served as controls.

**Results:**

We observed that elevated EPS8 expression in AML patients is associated with poor outcome. Knockdown of EPS8 significantly suppressed the survival of AML cells in vitro and in vivo. CP-EPS8-NLS interfered with EPS8-associated signaling and consequently exerted anti-AML activity. Importantly, CP-EPS8-NLS displayed anti-AML activity in various AML cell types, with diminished activity in PBMCs. CP-ESP8-NLS suppressed U937 cell proliferation, and injection of CP-EPS8-NLS exerted potent antitumor activity in the xenograft tumor models. A synergistic effect of CP-EPS8-NLS and chemotherapeutic agents was also observed in vitro and in vivo. Mechanistically, treatment of various AML cells with CP-EPS8-NLS downregulated the expression of EPS8 and its downstream pathways.

**Conclusions:**

The function of CP-EPS8-NLS is explained by the presence of a NLS in EPS8, which has been shown to induce nuclear translocation, consequently resulting in EPS8 overexpression. These results indicate that EPS8 is a potential target for AML treatment.

**Electronic supplementary material:**

The online version of this article (10.1186/s13046-018-0682-x) contains supplementary material, which is available to authorized users.

## Background

Despite advances in modern chemotherapy, the prognosis of patients with acute myeloid leukemia (AML) has remained poor and little progress has been made to improve long-term outcome of these patients. The American Cancer Society estimates that 21,380 new AML cases were diagnosed and approximately 10,590 deaths from this disease occurred in 2017 [1]. The long term, disease-free survival of AML patients under age 60 remains approximately 40% [2]. Therefore, new approaches are needed if further improvement in the outcome for AML patients is to be achieved.

EPS8 (epidermal growth factor receptor (EGFR) pathway substrate no.8) was first known as a vital substrate for EGFR kinase [3]. EPS8 is efficiently phosphorylated by various tyrosine kinases, both of the receptor (RTK) and non-receptor type [4] and is a typical signaling protein of 97 kDa, containing a phosphotyrosine binding protein (PTB) domain, a Src homology 3 (SH3) domain and a sterile alpha-pointed (SAM-PNT) domain [4]. Further studies of EPS8 have revealed the existence of two additional functional regions. A C terminal effector region, extending from amino acids (aa) 641 to 822, is thought to interact with Sos-1 and subsequently activate Rac specific GEF activity [5]. The other region, encompassing amino acids 298 to 362, provides a binding surface for the JXM region of EGFR (JMB) [6]. Importantly, a nuclear localization signal (NLS) is also in this region. Elevated EPS8 expression levels have been found in various solid tumors [7–10] and several hematological malignancies [11]. Studies have shown that EPS8 is critical in tumorigenesis, proliferation, invasion and metastasis [12–15]. Our previous review has provided a comprehensive picture of the role of EPS8 in different tumor biological behaviors [16]. Therefore, EPS8 might represent a novel potential target for cancer therapy.

The studies of EPS8 in hematological malignancies are limited. Elevated EPS8 expression was correlated with worse outcome in infant acute lymphoblastic leukemia (ALL) based on gene expression profiles (From a Children’s Oncology Group study) [11]. We have indicated that EPS8 may be a valuable clinical biomarker for assessing the outcome of ALL patients [17]. Our previous work showed that EPS8 was overexpressed in AML patients, and the expression level of EPS8 was correlated to the complete remission rate of AML patients treated with chemotherapy [18]. The 298–362 aa domain of EPS8 contains a nuclear localization signal. The release of EPS8 from tyrosine kinases makes the nuclear targeting signal available to the intracellular molecular machinery responsible for nuclear translocation. R Carbone et al. observed that a fraction of EPS8 is indeed translocated to the nucleus, resulting in increased EPS8 expression [6]. Ectopic EPS8 expression enhances mitogenic signals, eventually resulting in carcinogenesis.

This study consists of two major parts. First, we found that the MAPK/Erk pathway and PI3K/Akt pathways may play critical roles in EPS8-mediated induction of AML cell proliferation, anti-apoptosis and chemosensitivity in vitro and in vivo. Second, to overcome the limitations of currently available inhibitors for AML treatment, we developed an effective anti-AML peptide (CP-EPS8-NLS) derived from the 298–362 aa domain that specifically mimics the NLS of EPS8. Our findings demonstrate the efficacy of CP-EPS8-NLS in potently repressing AML cell lines both in vitro and in vivo, suggesting a novel therapeutic strategy for inhibition of the NLS of EPS8 in AML therapy.

## Methods

### Cell lines and culture conditions

The acute promyelocytic leukemia cell lines HL-60 and NB4, acute monocytic leukemia cell line THP-1, acute myelomonocytic leukemia cell line U937, acute erythrocytic leukemia cell line TF1α and acute myelogenous leukemia cell line KG1α were cryopreserved in the Hematological Laboratory of Zhujiang Hospital (Guangzhou, China). HL-60, NB4, TF1α and THP-1 cell lines were purchased from the cell bank of Sun Yet-san University (Guangzhou, China); the source of these cells was ATCC. U937 cell line was purchased from ATCC. The KG1α and HL-60/ADR cell lines were kindly provided by Tianjin Institute of Hematology (Tianjin, China). Normal PBMCs were obtained from 5 unrelated healthy donors at Southern Medical University (Guangzhou, China). All cell lines and the PBMCs were incubated in RPMI 1640 medium (Invitrogen, Carlsbad, CA) supplemented with 10% fetal bovine serum at 37 °C with 5% CO_2_.

### EPS8 GEO expression profile in AML cells

The expression profiles of AML patients from the GSE13159 dataset, containing bone marrow samples from 501 AML patients at diagnosis and 72 healthy volunteers, were generated on Affymetrix Gene Chip HG-U133A arrays (Affymetrix, Santa Clara, CA, USA), and were extracted from CEL files using RMA normalization procedure and custom CDF annotation package. AML samples from GSE12417 and The Cancer Genome Atlas (TCGA) were used to test association between EPS8 expression and AML patient outcomes.

### Creation and characterization of stable EPS8 knockdown cell lines

EPS8 expression was stably knocked down in U937 and KG1α cells via RNA interference. The annealed oligonucleotide fragments encoding short hairpin transcripts corresponding to EPS8 were as follows: TAGTGATTCAGGAGTGGAA and AACTTCTAATCGCCATATA. The non-targeting empty plasmid was used as the control shRNA plasmid. According to the manufacturer’s instructions, U937 and KG1α cells (2 × 10^5^/well in six-well plates) were separately transfected separately with control shRNA plasmid or the EPS8 shRNA plasmid using Lipofectamine 2000 reagent (Invitrogen). After the dilution culture was limited under selection with puromycin, several clones in each transfection group were selected for further experiments and designated as U937/NC, U937/sh1, U937/sh2, KG1α/NC and KG1α/sh1.

### RT^2^profiler™ PCR assay in KG1α/sh1 and KG1α/NC cells

An RT^2^profiler™ PCR assay (SuperArray, SABiosciences, a QIAGEN Company) was used to profile the expression of 84 EGF/PDGF signaling-specific genes plus 5 housekeeping genes according to the manufacturer’s protocol. The process in detail was described in previous work [19].

### Peptide synthesis

CP-EPS8-NLS, mutated CP-EPS8-NLS and penetratin were synthesized by the Chinese Peptide Company (Hangzhou, China). Peptides purity was greater than 95%. The peptides were dissolved in deionized water at a final concentration of 10 mg/ml and stored at − 20 °C until further use.

### Cell viability assays

AML cell lines and normal PBMCs were plated in a 96-well plate (5 × 10^3^ cells/well) and incubated for 24 h before treatment. All cells were then incubated with different concentrations of CP-EPS8-NLS (0, 35, 70, 105, 140 or 175 μM) for 24 h. U937 cells were also treated with mutated CP-EPS8-NLS and penetratin as controls. After treatment, 10 μl of CCK-8 reagent (Dojindo Laboratories, Japan) was added to each well, and cells were incubated for 3 h at 37 °C and 5% CO_2_. The optical density (OD) was analyzed at 450 nm. The data obtained are presented as percentage viability in best-fit (linear) dose response curves.

### Soft agarose cloning assay

Low-melting agarose was dissolved in pure water at 1.2 and 0.7%, sterilized using an autoclave, and then warmed at 42 °C in a water bath. Then, 1 ml of 2× RPMI 1640 was transferred to each well of a 6-well plate, and 1 ml of 1.2% agarose was added. After these two solutions were mixed, 500 μl of 0.7% agarose and 500 μl of RPMI 1640 containing 500 cells were pipetted into each well and treated with CP-EPS8-NLS (0, 35, 70, 175 μM) on the following day. Two weeks later, the colony number was determined.

### Cellular distribution of CP-EPS8-NLS in U937 cells

To examine the membrane penetration ability and the distribution of CP-EPS8-NLS, mutated CP-EPS8-NLS and penetratin in AML cells. Fluorescein isothiocyanate (FITC) was conjugated to the N-terminus of these peptides to form FITC-conjugated peptides. U937 cells (2 × 10^5^ cells per plate) were placed in confocal microscope observation wells that had been pretreated with polylysine. Then, cells were treated with FITC-conjugated peptides (40 μM) in 1 ml medium of culture for 4 h and the cells were stained with propidium iodide (PI) for 20 mins to exclude the possibility that peptides penetrate dying cells and then washed twice with PBS. The cells were fixed for 30 mins and stained with DAPI (which produces blue fluorescence after binding to dsDNA). Cells were rinsed three times with PBS, and the fluorescence distribution was analyzed with a confocal laser scanning microscope (LSM 880 with Airyscan).

### Analysis of apoptosis and cell cycle

U937, KG1α, HL-60, THP-1 and TF1α cells were seeded at 1 × 10^5^ cells/well in 6-well plates in serum-containing media; cells were cultured for 12 h before treatment. CP-EPS8-NLS was added at concentrations ranging from 0 to 175 μM and incubated at 37 °C and 5% CO_2_ for 24 h and 48 h respectively in KG1α. U937, KG1α, HL-60, THP-1 and TF1α cells were added at 0 or 70 μM CP-EPS8-NLS for 24 h. CP-EPS8-NLS treated AML cells were collected and washed by PBS, suspended in binding buffer according to the manufacturer’s protocol (BD, Annexin-V-APC & PI Apoptosis Detection Kit). Cells were analyzed with CellQuest software and each measurement was repeated three times to ensure reproducibility. For cell cycle analysis, AML cells were cultured for 12 h before treatment. CP-EPS8-NLS was added to a final concentration of 0 or 70 μM and incubated at 37 °C and 5% CO_2_ for 24 h. Cells were collected, washed with PBS, and suspended in RNase A and PI for 1 h in the dark. Samples were analyzed on a FACSCalibur Flow Cytometer (Becton Dickinson, New Jersey, USA).

### Chemicals

Daunorubicin (DNR), cytarabine (Ara-c), adriamycin (ADR) and perifosine were purchased from Selleckchem, dissolved in RPMI 1640 medium at a final concentration of 10 mg/ml and stored at − 20 °C.

### Determination of combination index values and Chou-Talalay analysis

KG1α and U937 cells were seeded at 5 × 10^3^ cells/well in 96-well plates for 24 h before treatment. Cells were treated with CP-EPS8-NLS and/or chemotherapeutic agents (DNR, Ara-c or ADR) at 37 °C and 5% CO_2_ for 24 h. Then, cell viability was measured using a CCK-8 assay. The assessment of synergy was performed using CompuSyn software. The combination index (CI) theorem of Chou-Talalay offers a quantitative definition for additive effect (CI = 1), synergism (CI < 1) or antagonism(CI > 1) in drug combinations [20]. Isobolograms were also used to better investigate the combination effects.

### Western blot analysis

All prepared cells were homogenized in protein lysate buffer, and debris was removed by centrifugation at 12,000 g for 10 min at 4 °C. The protein concentrations were determined using a Bradford protein assay kit (Beyotime, China). After addition of loading buffer, protein samples were electrophoresed, transferred to PVDF membranes (0.2 μm; Millipore, Bedford, MA), and subsequent blocked. The membranes were immunoblotted with rabbit anti-human primary antibody overnight at 4 °C. Antibodies to EPS8, Erk, p-Erk, Akt, p-Akt (473), p-Akt (450), p-Akt (308), p-Stat3, mTOR, p-mTOR, p38 MAPK, p-GSK3β, p-cRaf, Cyclin E, bcl-2 and GAPDH were obtained from Cell Signaling Technology. After three washes with TBST, the blots were incubated with horseradishperoxidase (HRP)-conjugated secondary antibodies at room temperature for 1 h, and the HRP signal was detected using enhanced chemiluminescence (Pierce Biotechnology, Rockford, IL, USA).

### In vivo study

All animal experiments complied with Southern Medical University’s Policy on the Care and Use of Laboratory Animals. Five-week-old athymic BALB/c nu/nu female mice (14–16 g) purchased from the experimental animal center of Southern Medical University (Guangzhou, China) were used for in vivo experiments. Animals were housed at a constant room temperature with a 12 h light/12 h dark cycle and fed a standard rodent diet and water. U937 cells were harvested and injected subcutaneously (5 × 10^6^ cells in 100 μl of PBS) into mice. U937-injected mice were treated with CP-EPS8-NLS at the dose of 50 mg/kg body weight or with PBS as a control via intraperitoneal (i.p.) injection every other day. In addition, KG1α cells were harvested and injected subcutaneously (1 × 10^7^ cells in 100 μl of PBS) into mice. KG1α-injected mice were treated with CP-EPS8-NLS (50 mg/kg) and/or DNR (20 mg/kg) every other day. Mutated CP-EPS8-NLS and PBS were injected as controls. The maximum tumor volume was not allowed to exceed 3000 mm^3^. At the end of the experiment, the animals were sacrificed, and the tumors were removed. The tumor volumes were determined by measuring tumor length (L) and width (W) and calculating the volume (V = 0.5 × L × W^2^) [21].

### Statistical analysis

Statistical significance was evaluated using SPSS 11.0 software. *P* < 0.05 was considered statistically significant. * Represents *P* < 0.05, ** represents *P* < 0.01, and *** represents *P* < 0.001.

## Results

### EPS8 expression profiles in AML patients

**Fig. 1:**
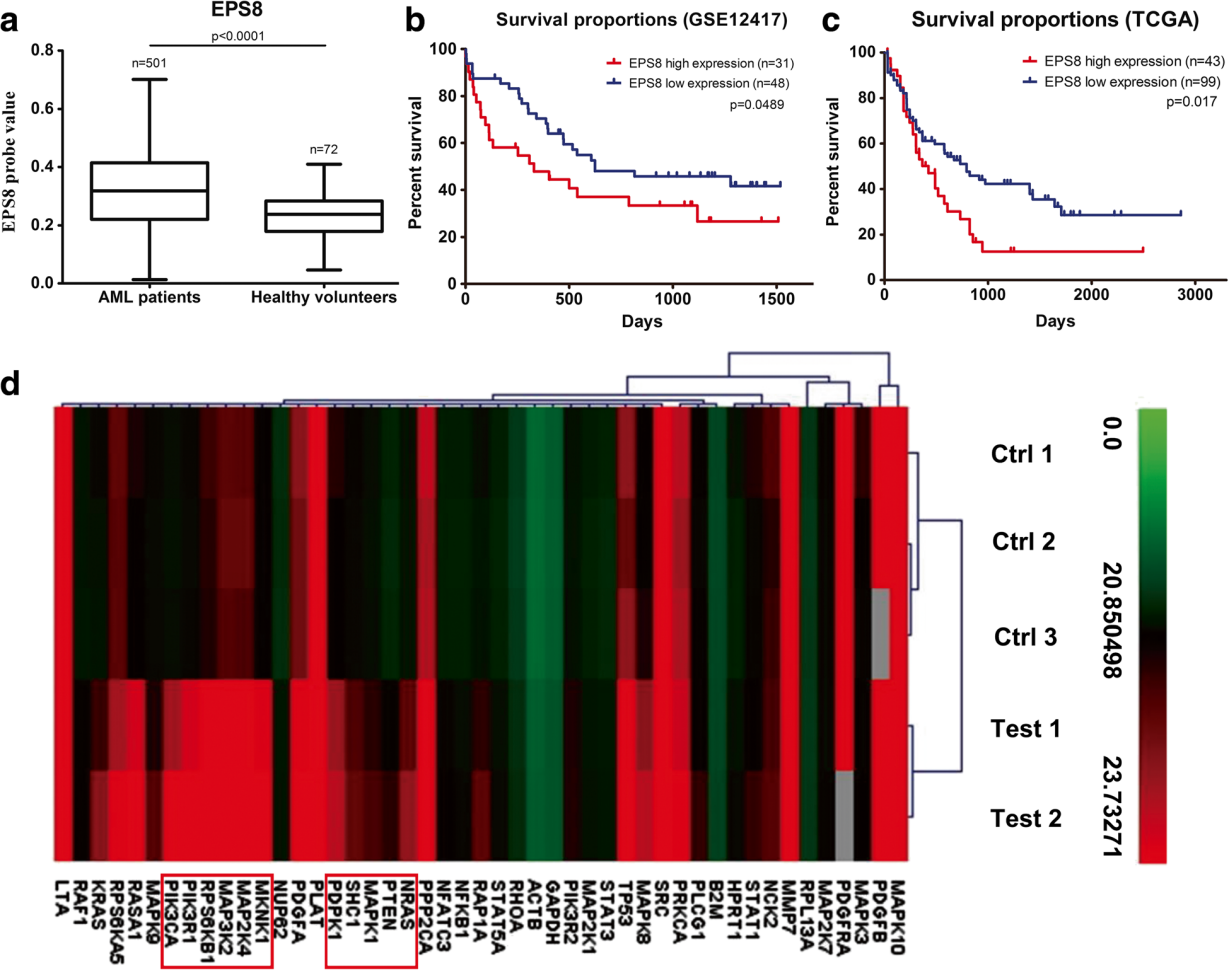
EPS8 expression in AML patients. **a** Box plot presenting the median level of EPS8 expression in 501 AML patients at diagnosis and in 72 healthy volunteers from GSE13159 dataset. **b** Kaplan-Meier estimated curves represent the percentage of overall survival of AML patients from GSE12417 dataset. High EPS8 expression was associated with poor overall survival (median survival, 330 vs 624 days). **c** Kaplan-Meier estimated curves represent the percentage of overall survival of AML patients from TCGA database. High EPS8 expression was associated with poor overall survival (median survival, 424 vs 792 days). **d** Changes in important EGF/PDGF signaling pathway targets after EPS8 knockdown analyzed with an RT^2^profiler™ PCR assay in KG1α cells

To assess the correlation between EPS8 expression level and the clinical outcome of AML patients, we analyzed the whole transcriptional profiles of the GSE13159 dataset and found higher expression levels (*p* < 0.0001) in bone marrow samples from AML patients (*n* = 501) than in healthy volunteers (*n* = 72) (Fig. 1a). This prompted us to test whether an association could be established between EPS8 expression and patient clinical outcome. In total, 79 AML samples from GSE12417 and 142 AML samples from TCGA were extracted for prognostic analysis. EPS8 expression values above the average value were defined as EPS8 high expression and values below the average value were defined as EPS8 low expression. A significant association was identified between EPS8 expression and OS. High EPS8 expression levels (above the average value) were associated with poor prognosis, as shown by the Kaplan-Meier survival curve presented in Fig. 1b and c.

### EPS8 knockdown induces specific changes in gene expression

**Fig. 2:**
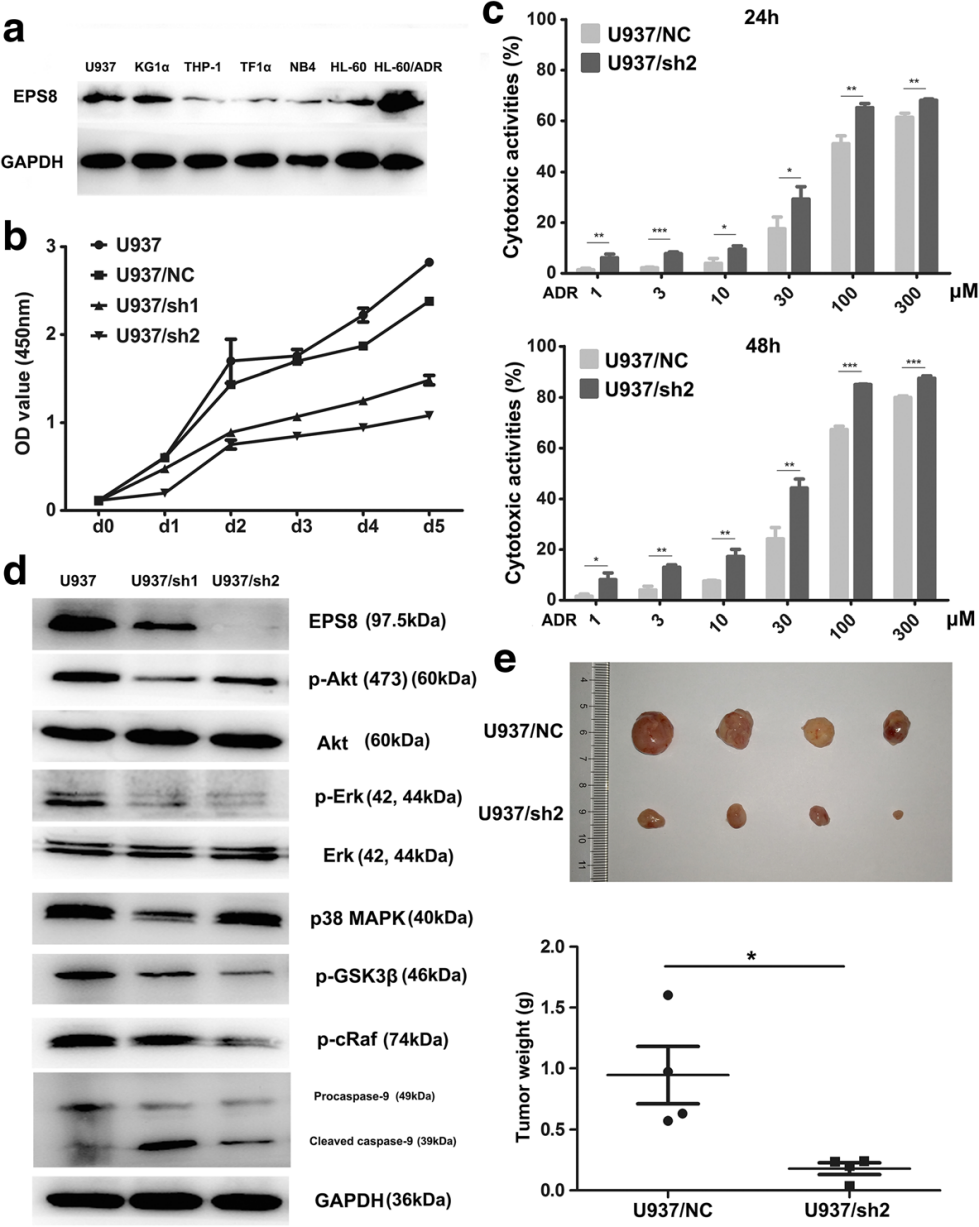
The ability of EPS8 to influence the AML cells survival. **a** Western blot assay of EPS8 in six AML cell lines and an adriamycin-resistant AML cell line. **b** Cell survival assay in EPS8 shRNA-infected U937cells compared with NC shRNA-infected and parental cells on days 0 to 5. U937/NC and U937 cells nearly filled the well by 5th day. **c** Chemosensitivity of U937 cells transfected with shRNA2 after ADR treatment for 24 and 48 h. **d** U937 cells transfected with shRNA1, shRNA2 and NC shRNA were subjected to western blot analysis for EPS8, Akt/pAkt, Erk/p-Erk, p38 MAPK, p-GSK3β, p-cRaf, Caspase-9 and GAPDH expression. **e** Representative images of tumors extracted from nude mice following injection of 5 × 10^6^ NC shRNA-infected cells and Eps8 shRNA-infected cells. The weights of the excised tumors

To screen the expression levels of EPS8 in hematological malignancy cell lines, we performed a western blot analysis. The results indicated EPS8 overexpression in KG1α and U937 cell lines and lower EPS8 expression in the four AML cell lines (NB4, HL-60, THP-1 and TF1α) (Fig. 2a). Crucially, EPS8 was highly overexpressed in an Adriamycin resistant HL-60 cell line (HL-60/ADR) compared with its expression in HL-60 cell line (Fig. 2a). To further characterize the action of EPS8 in AML cells, we knocked down EPS8 expression in KG1α cells, performed gene expression profiling, and compared the results with the profile of vehicle-treated KG1α cells. An RT^2^ profiler™ PCR assay was performed to analyze changes in important EGF/PDGF signaling pathway targets. In total, 82 unique genes were downregulated and 8 were upregulated after EPS8 knockdown. The hierarchical clustering results shown in Fig. 1d and Additional file 1: Figure S1 demonstrated that EPS8 knockdown (− 4.46-fold) remarkably downregulated the expression of PI3K/Akt- and MAPK/Erk-associated pathway targets: PIK3C1 (− 10.27-fold), PIK3R1 (− 10.42-fold), PIK3R2 (− 2.09-fold), PTEN (− 5.24-fold), MAP3K2 (− 12.42-fold), MAP2K4 (− 5.16-fold), MKNK1 (− 6.22-fold) and MAPK8 (− 3.16-fold).

### EPS8 knockdown inhibits AML cells survival in vitro and in vivo

We introduced an EPS8 shRNA vector into U937 and KG1α cells that exhibited high EPS8 protein expression. We investigated whether shRNA-mediated EPS8 knockdown inhibited AML cells survival. The effects of EPS8 knockdown on proliferation, apoptosis and chemosensitivity were investigated. Compared with control cells, the EPS8-silenced cells had lower levels of cell proliferation than the corresponding vector control and normal control cells (Fig. 2b and Additional file 2: Figure S2B). Colony formation assays showed that EPS8 knockdown led to fewer and smaller colonies in KG1α or U937 cells, while control shRNA had no effect (Additional file 2). We further investigated the role of EPS8 in AML cells chemosensitivity. We observed that the sensitivity of U937 cells to ADR was significantly increased in U937/sh1 and U937/sh2 cells (Fig. 2c). Because EPS8 influences many genes involved in the development and progression of human cancers, we decided to examine whether EPS8 regulates targets associated with these processes in AML cell lines. The gene microarray results indicated that the PI3K/Akt and MAPK/Erk pathways were downregulated in KG1α cells stably transfected with shEPS8, and thus, to further characterize the underlying mechanism by which EPS8 promotes tumor growth, western blotting was used to identify altered pathways in cells treated with the specific PI3K/Akt inhibitor perifosine [22] (Additional file 2: Figure S2A) and in cells with EPS8 silenced by shRNA (Fig. 2d and Additional file 2: Figure S2D). The western blotting results confirmed that phosphorylated Akt and phosphorylated Erk, were downregulated after EPS8 knockdown in U937 cells. Tumorigenicity assays were performed by subcutaneous injection of EPS8 shRNA cells (U937/sh2) into nude mice, and NC shRNA cells were used as the control. Twenty eight days after injection, the average volume and weight of the U937/sh2 group were markedly reduced compared with the values in the U937/NC group (Fig. 2e). Taken together, these results suggest that EPS8 might be a novel positive regulator of AML cell survival in vitro and in vivo.

### Cp-EPS8-NLS

**Fig. 3:**
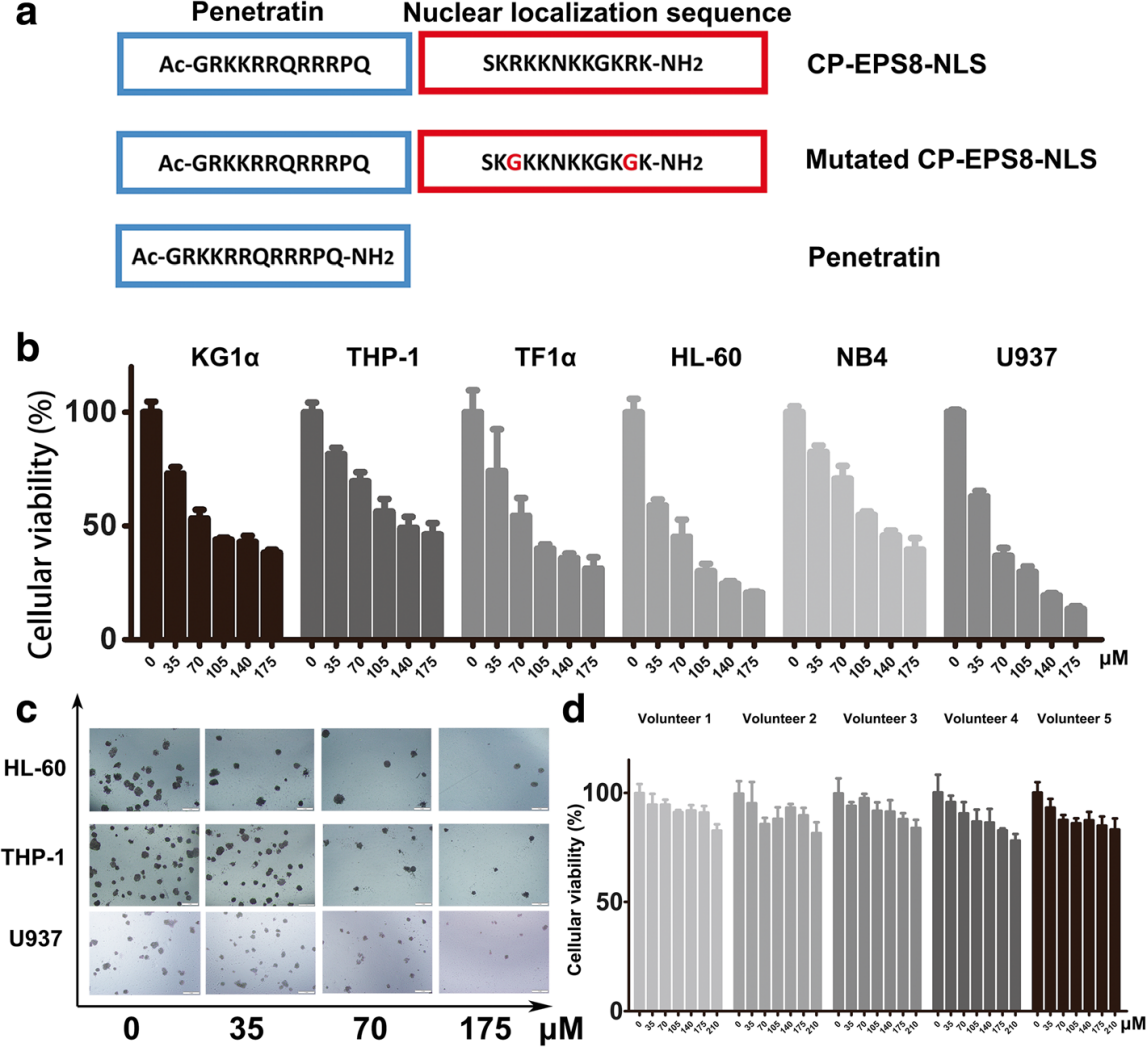
Characterization of CP-EPS8-NLS, a specific peptide that localizes to the nucleus. **a** Graphical representation showing the sequences of CP-EPS8-NLS, mutated CP-EPS8-NLS, and penetratin. **b** HL-60, NB4, THP-1, U937, TF1α and KG1α cells were treated for 24 h with increasing concentrations of CP-EPS8-NLS analyzed by CCK-8. **c** HL-60, THP-1 and U937 cells were seeded and treated with increasing concentrations of CP-EPS8-NLS for 2 weeks. Colonies were counted, and images were taken. **d** PBMCs from 5 unrelated healthy donors were treated with increasing concentrations of CP-EPS8-NLS for 24 h, and cell viability was analyzed via CCK-8 assay

CP-EPS8-NLS is a synthetic 24 amino acid peptide that was engineered to cross the cellular membrane and specifically interfere with the nuclear translocation of EPS8. The N-terminal end of CP-EPS8-NLS has a TAT sequence that facilitates cell penetration. The core nuclear localization sequence (SKRKKNKKGKRK) derived from the 298–310 aa domain serves a dual function: it can guide the EPS8 protein to the nucleus and modulate downstream activity. For control purposes, the peptides with a penetration domain alone and peptides in which key arginines was mutated to glycines were also synthesized to reduce the nuclear localization effect (Fig. 3a).

### CP-EPS8-NLS suppresses cell viability and AML cells proliferation

**Fig. 4:**
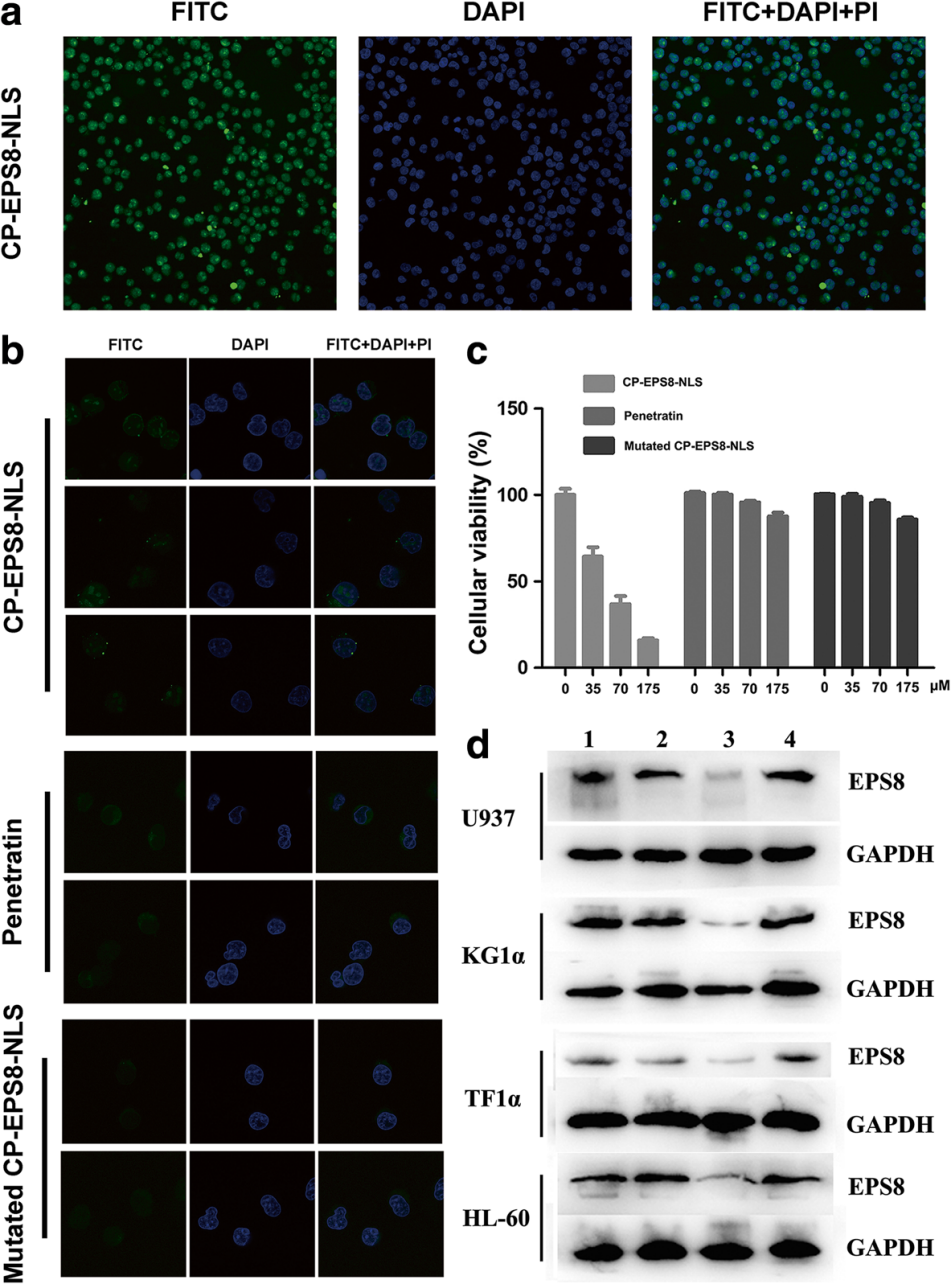
Intracellular distribution of CP-EPS8-NLS in U937 cells. **a** U937 cells (2 × 10^5^ cells per plate) were transduced with FITC-conjugated CP-EPS8-NLS (40 μM) in 1 mL of culture medium for 2 h and stained with PI and DAPI. Significant green fluorescence could be observed both in the nucleus and cytoplasm. **b** U937 cells were treated with either mutated CP-EPS8-NLS or penetratin. Green fluorescence was only observed in the cytoplasm. **c** U937 cells were treated with increasing concentrations of CP-EPS8-NLS, mutated CP-EPS8-NLS, and penetratin. After 24 h, a CCK-8 assay was performed. **d** Four AML cell lines were treated with 70 μM CP-EPS8-NLS, mutated CP-EPS8-NLS, and penetratin for 12 h and analyzed by western blot. CP-EPS8-NLS significantly decreased the expression of EPS8, while mutated CP-EPS8-NLS and penetratin did not (1. Control; 2. Mutated CP-EPS8-NLS; 3. CP-EPS8-NLS; 4. Penetratin)

Initially, to assess the activity of CP-EPS8-NLS, KG1α, U937, THP-1, TF1α, HL-60, NB4 and U937 cells were treated for 24 h with increasing concentrations of the peptide (0-175 μM). A dose-dependent anti-proliferative effect of CP-EPS8-NLS was determined using CCK-8 assays (Fig. 3b). To verify whether the effect of the peptide on survival is specifically related to the NLS and not to the cell-penetration domain, we treated U937 cells with CP-EPS8-NLS, mutated peptide or the penetration domain alone. In contrast with CP-EPS8-NLS, the mutated peptide and the penetration domain did not markedly suppress cell proliferation (Fig. 4c). We also examined the effects of CP-EPS8-NLS inhibition on colony formation using soft agar formation assays (Fig. 3c). The numbers of colonies decreased as the concentration of CP-EPS8-NLS increased. Moreover, CP-EPS8-NLS did not result in significant suppression of PBMCs from 5 unrelated healthy volunteers (less than 10%), suggesting that CP-EPS8-NLS possesses specificity toward AML cells (Fig. 3d). Collectively, these results indicate that CP-EPS8-NLS effectively suppresses cell survival and growth.

### CP-EPS8-NLS traverses the cell membrane and localizes in nucleus

The cell penetrating sequence in the N terminal of CP-EPS8-NLS allows the peptide to traverse the cell membrane and facilitates the nuclear localization function of the peptide. To confirm these activities, CP-EPS8-NLS, mutated CP-EPS8-NLS and penetratin were labeled with FITC and observed using laser scanning confocal microscopy. To observe the distribution of FITC-conjugated peptides, U937 cells were treated with FITC-labeled peptides (40 μM) for 2 h and observed under a laser scanning confocal microscope. As shown in Fig. 4a and b, strong, flaky blue fluorescence was observed in nuclei in all three groups, which revealed the nuclear outlines. Green fluorescence produced by FITC-conjugated CP-EPS8-NLS densely covered both the cytoplasm and the nucleus after treatment with CP-EPS8-NLS. In contrast, there was no green fluorescence in nuclei after treatment with mutated CP-EPS8-NLS or penetratin (Fig. 4b). Moreover, after cells were stained with PI for 20 mins, no red fluorescence was observed in any of three groups. However, red fluorescence was observed after treatment with CP-EPS8-NLS for 8 h (Additional file 3), which may be due to cytotoxic effect of CP-EPS8-NLS. These results indicate that CP-EPS8-NLS could successfully traverse the cell membrane and localize to the nucleus.

### CP-EPS8-NLS promotes apoptotic cell death and cell cycle arrest

**Fig. 5:**
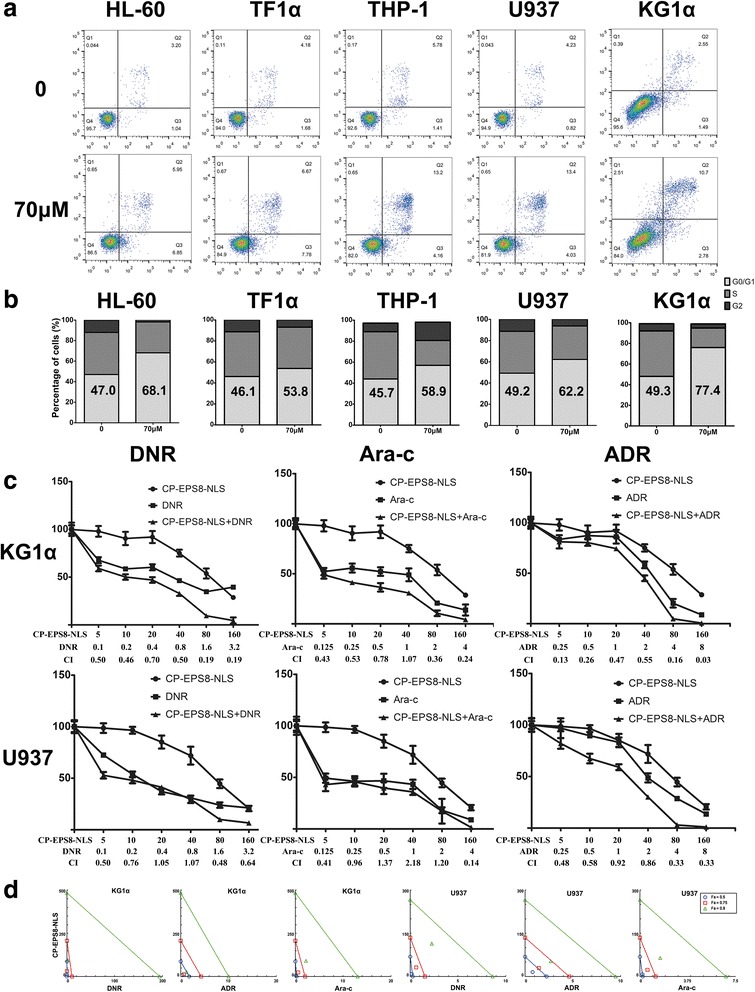
CP-EPS8-NLS potently suppresses human AML cell growth and increases chemosensitivity. **a** Flow cytometric analysis of Annexin V-FITC/PI–stained U937 cells treated with increasing concentrations of CP-EPS8-NLS for 24 h. **b** The effect of CP-EPS8-NLS on cell cycle: Following treatment of KG1α, U937, HL-60, TF1α, and THP-1 cells with CP-EPS8-NLS for 24 h and PI staining, cells were analyzed via flow cytometry and quantified. **c** Cells were cultured in the presence of daunorubicin (DNR; μmol/L), cytarabine (Ara-C; μmol/L), adriamycin (ADR; μmol/L) or CP-EPS8-NLS (μmol/L) alone or in combination at a fixed ratio. The combined treatment was highly effective in inducing cytotoxicity, as shown by CCK-8 assays at 24 h. The CI value for each data point was calculated with the appropriate software for dose effect analysis (CompuSyn). **d** Isobolograms were constructed using CompuSyn to further investigate the combination effects

To examine the apoptotic effect of CP-EPS8-NLS, KG1α cells were treated with increasing concentrations (0-175 μM) for 24 h and 48 h. As shown in Additional file 4: after treatment with CP-EPS8-NLS, the percentages of apoptotic cells increased in a dose- and time- dependent manner. Similar results were obtained in several other AML cell lines (Fig. 5a). We examined the effect of CP-EPS8-NLS on cell cycle in 5 AML cell lines. After overnight treatment, we observed an increase in the proportion of CP-EPS8-NLS treated cells in the G1/G0 phase of the cell cycle, with a corresponding decline in the proportion of G2/M phase cells (Fig. 5b).

### CP-EPS8-NLS synergizes with chemotherapeutic drugs

Previous work demonstrated that EPS8 decreases the chemosensitivity of cervical cancer patients and lung cancer patients [23, 24]. Our previous work also suggested that EPS8 was correlated with the complete remission rate of newly diagnosed AML patients after the first chemotherapy [18]. In support, knockdown of EPS8 significantly enhanced the sensitivity of AML cells to chemotherapeutic agents. Accordingly, we investigated whether CP-EPS8-NLS could synergize with drugs commonly used to treat AML patients. AML cell lines (U937 and KG1α) were incubated for 24 h with drugs alone, CP-EPS8-NLS alone, or CP-EPS8-NLS plus drugs at a fixed ratio (CP-EPS8-NLS/DNR, 50:1; CP-EPS8-NLS/Ara-c, 40:1; CP-EPS8-NLS/ADR, 20:1). CCK-8 assays were then performed. The Chou-Talalay analysis method [20] was used to determine additive (CI = 1), synergetic (CI < 1) or antagonistic (CI > 1) effects. Analysis of the results confirmed that CP-EPS8-NLS synergized acted synergistically with the abovementioned drugs (Fig. 5c and d).

### CP-EPS8-NLS downregulates the expression of EPS8 and downstream targets

**Fig. 6:**
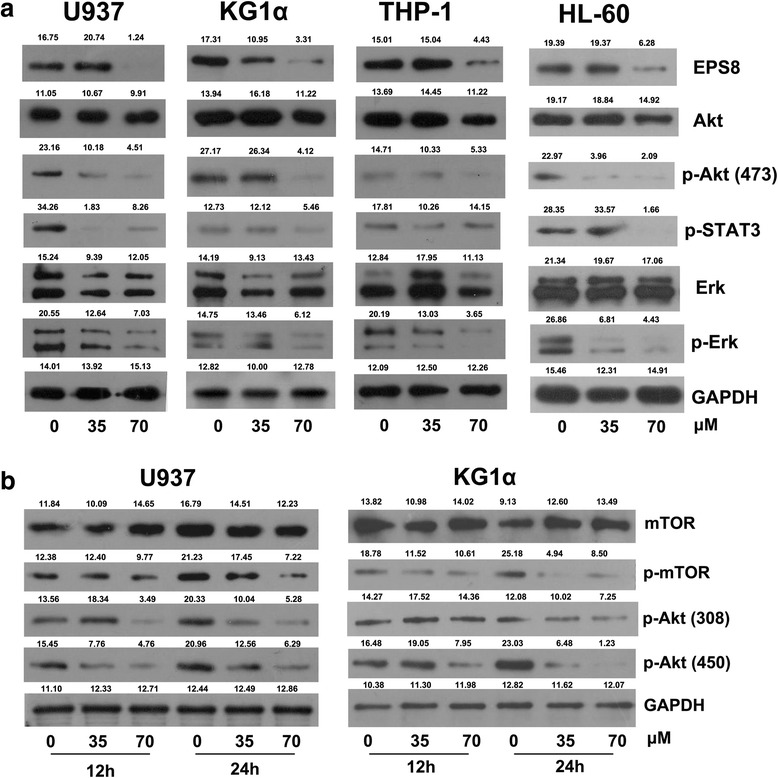
The inhibitory effects of CP-EPS8-NLS on EPS8 associated signaling. **a** U937, KG1α, HL-60 and TF1α cells were treated with increasing concentrations of CP-EPS8-NLS for 12 h, and analyzed by western blot to determine EPS8, Akt, p-Akt (473), p-STAT3, Erk and p-Erk levels. GAPDH expression was assessed to confirm equal protein loading. Densitometric analysis was performed using ImageJ software (NIH, Bethesda, MD; http://imagej.nih.gov/ij). **b** Western blot analyses of U937 and KG1α cells treated with 0, 35 or 105 μM CP-EPS8-NLS for 12 and 24 h were performed to examine mTOR, p-mTOR, p-Akt (308) and p-Akt (450) expression

Four AML cell lines (U937, KG1α, TF1α and HL-60) were treated with 70μΜ CP-EPS8-NLS, mutated CP-EPS8-NLS and penetratin for 12 h. The expression levels of EPS8 were significantly downregulated by CP-EPS8-NLS, while mutated CP-EPS8-NLS and penetratin did not significantly change EPS8 expression (Fig. 4d). Previous data support a role of EPS8 in amplifying receptor tyrosine kinase downstream signaling in several human solid tumors to promote proliferation and cell survival. Knockdown of EPS8 resulted in a robust downregulation of MAPK/Erk signals and a modest downregulation of PI3K/Akt signals in solid tumors. RT^2^ profiler™ PCR assay results showed that MAPK/Erk and PI3K/Akt signaling was significantly downregulated after silencing of EPS8. To further examine the inhibitory effect of CP-EPS8-NLS on downstream MAPK/Erk and PI3K/Akt signaling, we measured p-Akt, Akt, Erk, p-Erk, p-stat3, mTOR and p-mTOR protein expression in AML cell lines (U937, KG1α, TF1α and HL-60) after CP-EPS8-NLS treatment for 12 h. Phosphorylated Erk levels were inhibited in a dose-dependent manner in all four cell lines, while the total Erk levels were unaffected. Akt proteins (473, 308 and 450) phosphorylation levels were also significantly decreased in four cell lines after CP-EPS8-NLS treatment, while the total Akt levels were unchanged. After treatment with CP-EPS8-NLS, EPS8 expression levels in four AML cell lines were significantly reduced, and the reduction was correlated with downregulation of the MAPK/Erk and PI3K/Akt pathways (Fig. 6a and b). Western blotting analyses of U937 and KG1α cells treated with 0, 35 or 105 μM CP-EPS8-NLS for 12 or 24 h were performed to detect mTOR, p-mTOR, p-Akt (308) and p-Akt (450) levels. We noticed decreases in the abovementioned proteins in AML cells after CP-EPS8-NLS treatment (Fig. 5a and b).

### CP-EPS8-NLS inhibits progression of AML cells in vivo

**Fig. 7:**
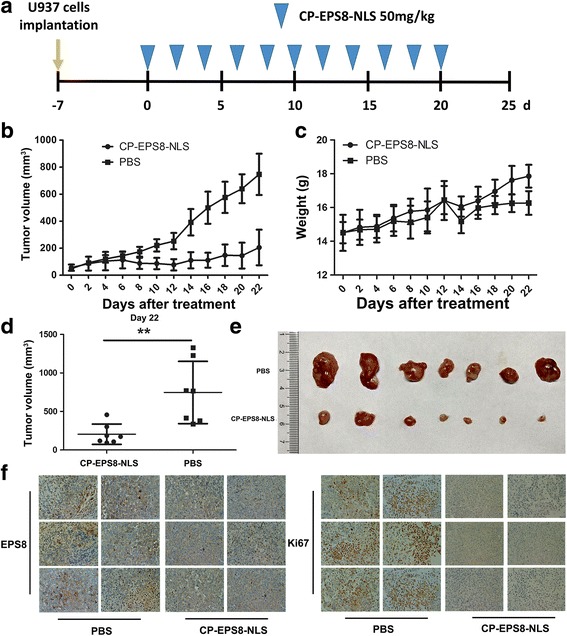
Antitumor activity of CP-EPS8-NLS in a U937 xenograft model. **a** U937 cells (5 × 10^6^ per flank) were injected into the right flank of mice. When the volume of tumors reached 100 mm^3^, the mice were randomly divided into two groups (7 mice in each group). Then, PBS or CP-EPS8-NLS were intraperitoneally injected into the mice every other day. **b** The volume of each tumor was measured every other day for 22 days. **c** Body weight of the mice. **d** The volume of each tumor was measured after tumor extraction from the mice at the experimental endpoint. **e** Image of tumors excised from mice after 22 days of treatment. **f** EPS8 and Ki67 in tumor tissues at the end of the experiments using IHC staining

To further validate the effect of CP-EPS8-NLS on the growth of AML cells, we employed a nude mouse xenograft assay by subcutaneously implanting U937 cells into nude mice (total *n* = 14). Once we detected palpable tumors, we randomized pairs of mice to receive either CP-EPS8-NLS at 50 mg per kg body weight (*n* = 7) or PBS solution (*n* = 7). Mice were sacrificed when one or more controls reached the maximum tumor burden permitted in our mouse protocol. Compared with the control treatment, CP-EPS8-NLS potently lowered AML tumor sizes (Fig. 7b-e). Wealso investigated the cell proliferation rate through Ki67 staining of the tumor samples. The CP-EPS8-NLS-treated group exhibited a markedly reduced number of Ki67-positive cells compared with that in the PBS control group (Fig. 7f), indicative of a decrease in cell proliferation. Consistent with the results at the cellular level, CP-EPS8-NLS decreased the abundance of EPS8 in the tumor samples (Fig. 7f). There was no evidence of toxicity based on behavioral, macroscopic or microscopic assessments (Fig. 7e).

### CP-EPS8-NLS synergizes with chemotherapeutic drugs in vivo

**Fig. 8:**
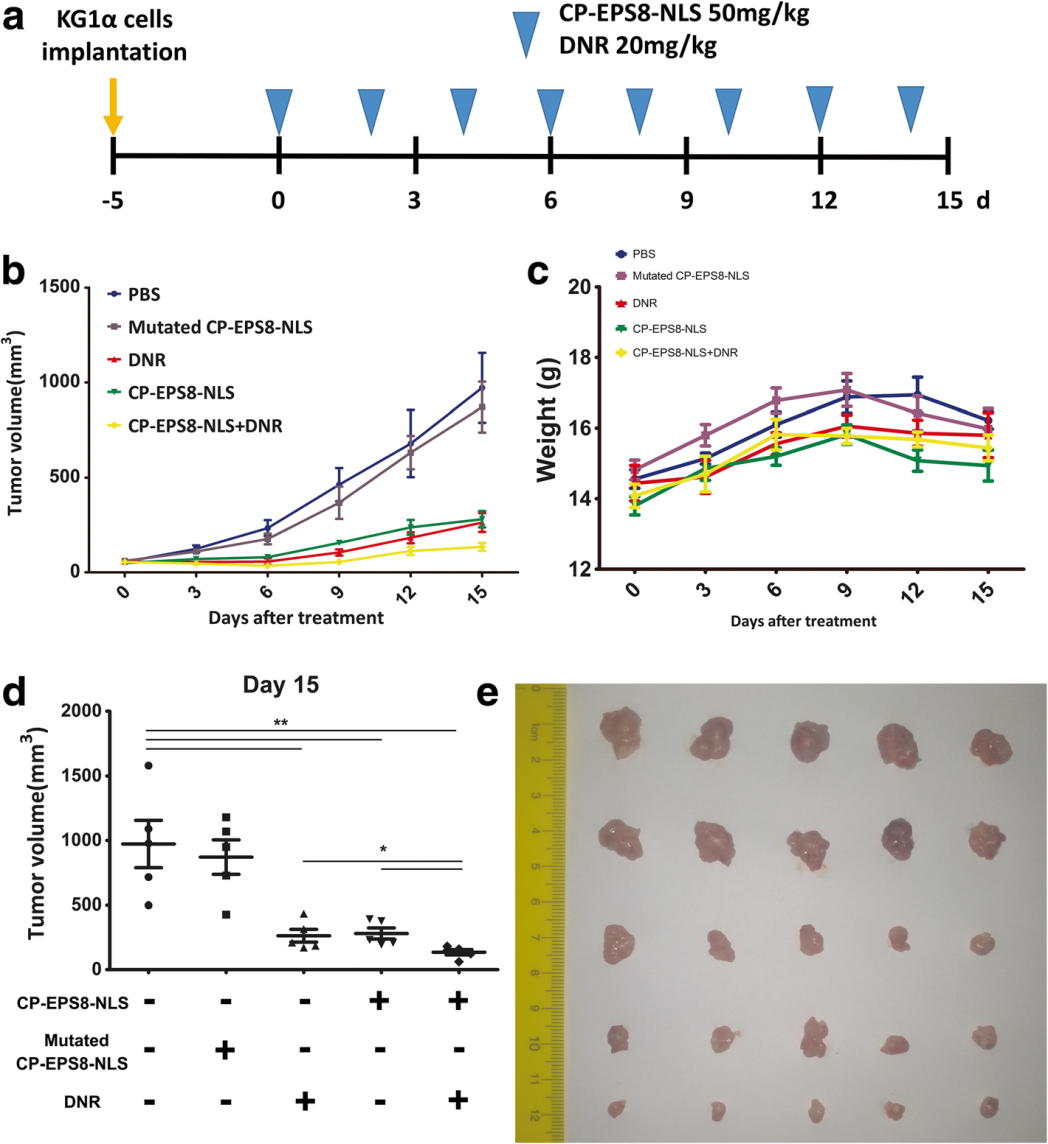
Synergistic effect of CP-EPS8-NLS with chemotherapeutic agents in a KG1α xenograft model. **a** KG1α cells (1 × 10^7^ per flank) were injected into the right flank of mice. When the volume of tumors reached 100 mm^3^, the mice were randomly divided into five groups (5 mice in each group). Then, CP-EPS8-NLS, DNR or CP-EPS8-NLS combined with DNR were intraperitoneally injected into the mice every other day. Mice injected with mutated CP-EPS8-NLS or PBS were used as controls. **b** The volume of each tumor was measured every 3 days for 15 days. **c** Body weight of the mice. **d** The volume of each tumor was measured after extraction from mice at the experimental endpoint. **e** Image of tumors excised from mice after 15 days of treatment

To further validate the synergistic effect of CP-EPS8-NLS with chemotherapeutic agents in vivo, we employed a nude mouse xenograft assay by subcutaneously implanting KG1α cells into nude mice (total = 25). Nude mice were treated with CP-EPS8-NLS (50 mg/kg), DNR (20 mg/kg) or CP-EPS8-NLS combined with DNR when we detected palpable tumors. PBS and mutated CP-EPS8-NLS (50 mg/kg) were treated as the control groups. The tumor volumes at the terminal point were smaller in the combined group than in both the CP-EPS8-NLS and DNR groups (Fig. 8). Moreover, mutated CP-EPS8-NLS did not decrease the tumor volume (Fig. 8).

## Discussion

EPS8 is important in regulating the development and progression of many human cancers. However, the role of EPS8 in hematological malignancies has not been clarified in detail. Elevated EPS8 expression is correlated with worse outcome in ALL patients. In our previous work, using real-time quantitative PCR to detect EPS8 RNA in bone marrow samples from AML patients, we found that EPS8 may have a clinical significance in monitoring (minimal residue disease) MRD that may lead to relapse. EPS8 is an important signaling molecule that integrates multiple pathways, and it controls the Ras-Raf-MEK-Erk signaling cascade, which plays a crucial role in regulating cellular processes including differentiation, proliferation, survival and apoptosis [25–27]. Moreover, formation of the EPS8–Abi1–Sos1 complex recruits to the activated RTKs, leading to Ras activation and integration with PI3K. PIP3, product of PI3K, is thus recruited and activates the PI3K/Akt pathway [28]. Accumulating evidence has shown the role of the PI3K/Akt pathway in the mechanisms of EPS8 associated tumor proliferation, survival and drug resistance by activating downstream targets, such as FOXM1, mTOR, MMP-9 and caspase-9 [15, 29]. Many studies have confirmed that aberrant PI3K/Akt/mTOR signaling is significantly related to progression of various solid tumors and hematological malignancies [30–32], and we found that EPS8 expression level were downregulated after the Akt signals were blocked (Additional file 1). We then used a lentivirus-based RNAi system to knock down EPS8 expression in AML cell lines. EPS8 attenuation suppresses the growth ability of AML cells and increases the sensitivity of AML cells to chemotherapy drugs. Our data indicated that EPS8 expression is critical for AML cell growth and proliferation in vitro and in vivo (Fig. 2). EPS8 is possibly involved in the progression and chemosensitivity of AML patients. In this work, we observed that U937/sh2 cells were more sensitive to chemotherapeutic agents (Fig. 2c). We hypothesized that inhibiting EPS8 overexpression or interfering with EPS8 associated signal transduction could eventually inhibit carcinogenesis of AML cells.

Previous studies have suggested that targeted inhibitors interacting with the NLS residues of tumor associated protein can block the nuclear localization ability of proteins in cancer cells, which is crucial for cell cycle progression and has a cellular inhibitory effect [33]. Here, we show that a novel, synthetic, cell-penetrating peptide derived from nuclear localization sequence of EPS8 induces apoptosis in a broad range of AML types, including acute promyelocytic leukemia (HL-60 and NB4 cells), acute monocytic leukemia (THP-1 cells), acute myelomonocytic leukemia (U937 cells), acute erythrocytic leukemia (TF1α cells) and acute myelogenous leukemia (KG1α cells). We observed the synergistic killing effect of CP-EPS8-NLS combined with therapeutic agents (DNR, Ara-c and ADR). Moreover, CP-EPS8-NLS successfully localized in the nucleus and downregulated the overexpression level of EPS8 in AML cells (directly or indirectly). Western blot assays showed downregulation of p-Akt and p-mTOR after CP-EPS8-NLS treatment. These results were compatible with an previous study demonstrating that activation of PI3K/Akt pathway could induce drug resistance of AML blasts in a PI3K/Akt/mTOR dependent manner [34]. Phosphorylation of Erk was also down-regulated while the total Erk levels were unchanged. These results verify the inhibitory effects of EPS8 associated AML signal transduction after CP-EPS8-NLS treatment. The in vivo studies provide primary evidence that CP-EPS8-NLS can selectively inhibit the AML cells growth in xenograft nude mouse models. EPS8 overexpression levels were also downregulated in tumor samples from nude mice. Another attractive feature of CP-EPS8-NLS is that it does not significantly suppress the viability of PBMCs from healthy donors. With respect to specificity, the penetratin peptide and a peptide in which key basic residues (lysines) in CP-EPS8-NLS were mutated showed no or little cytotoxic activity in viability studies, and neither peptides downregulated EPS8 expression. Mutated CP-EPS8-NLS also served as a control group in vivo. The fact that EPS8, a tumor associated protein, is overexpressed in AML cells, may indicate cause a state of cellular EPS8 dependency in the sense of oncogene addiction. The NLS of EPS8 may be responsible for nuclear translocation and further activate mitogenic signaling, which induces EPS8 overexpression. Thus, the selective response of AML cells to CP-EPS8-NLS treatment might be a consequence of a CP-EPS8-NLS mediated loss of the nuclear localization function of EPS8 and subsequent downregulation of EPS8.

## Conclusions

Taken together, our findings revealed the effect of CP-EPS8-NLS in inhibiting AML cells from responding to EPS8 induced activation and suppressing leukemia cells in vitro and in vivo, which demonstrates the potential of EPS8 targeting in leukemia therapies. In particular, the NLS of EPS8 may become a new target for further inhibitor design to interfere with EPS8 dependent AML progression.

## Additional files

Additional file 1: Changes in EGF/PDGF signaling pathway targets analyzed with a RT^2^ profiler™ PCR assay. **A** Array layout of the RT^2^ profiler™ PCR assay. **B** Changes in EGF/PDGF signaling pathway targets in KG1α/sh1 cells compared with those in KG1α/NC cells. (TIFF 6966 kb)

Additional file 2: The ability of EPS8 to influence AML cells survival. **A** Changes in EPS8 expression levels after treatment with increasing concentrations of the Akt inhibitor (perifosine) (0 to 40 μM) for 12 and 24 h analyzed by western blot. **B** Proliferation ability of KG1α cells after EPS8 knockdown. **C** Colony formation analysis in parental U937 cells or KG1α cells compared with shRNA1- and NC shRNA-infected cells. **D** Changes in EPS8 associated signaling pathways after EPS8 knockdown analyzed by western blot. (TIFF 7639 kb)

Additional file 3: U937 cells treated with CP-EPS8-NLS for 8 h and observed under a laser confocal scanning microscope. (TIFF 7912 kb)

Additional file 4: Percentage of apoptotic KG1α cells after CP-EPS8-NLS treatment for 24 and 48 h. (TIFF 4126 kb)

## Abbreviations

ADR: Adriamycin
AML: Acute myeloid leukemia
Ara-c: Cytarabine
DNR: Daunorubicin
EGFR: Epidermal growth factor receptor
EPS8: Epidermal growth factor receptor pathway substrate no.8
IHC: Immunohistochemistry
NLS: Nuclear localization signals/sequence
RTK: Receptor tyrosine kinase
