# Correction: A synthetic cell-penetrating peptide derived from nuclear localization signal of EPS8 exerts anticancer activity against acute myeloid leukemia

**DOI:** 10.1186/s13046-022-02446-7

**Published:** 2022-07-30

**Authors:** Yiran Chen, Xiaoling Xie, Anqin Wu, Lei Wang, Yuxing Hu, Honghao Zhang, Yuhua Li

**Affiliations:** Department of Hematology, Zhujiang Hospital, Southern Medical University, No. 253 GongyeDadaoZhong, Guangzhou, Guangdong 510282 People’s Republic of China

**Correction: J Exp Clin Cancer Res 37, 12 (2018)**

**https://doi.org/10.1186/s13046-018-0682-x**

Following publication of the original article [1], an error was identified in Fig. 7; specifically:

- Figure 7f: slice (PBS-4) was occupied by a duplicate slice (PBS-6); the correct image is now used.

**Fig. 7:**
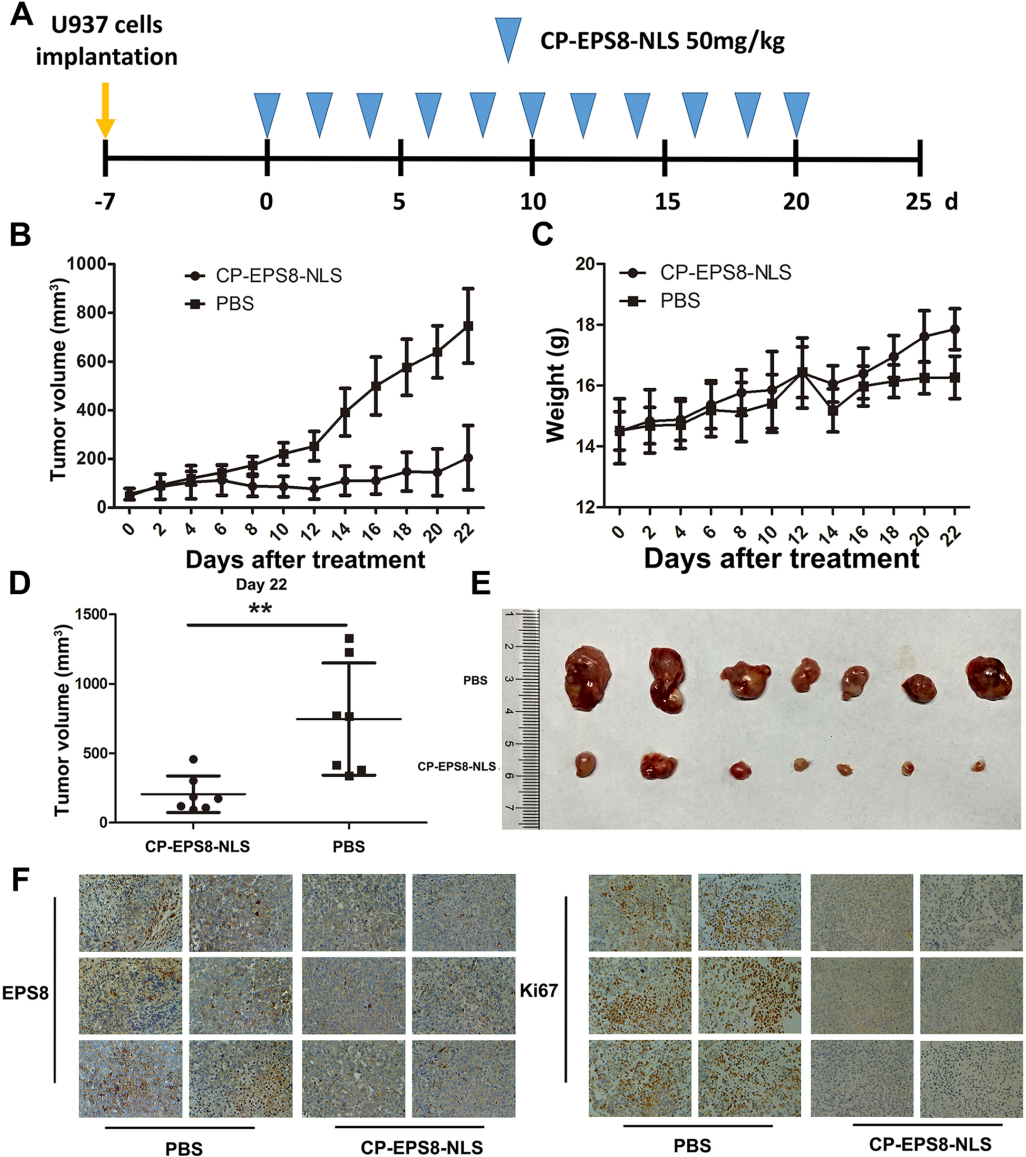
Antitumor activity of CP-EPS8-NLS in a U937 xenograft model. **a** U937 cells (5 × 10^6^ per flank) were injected into the right flank of mice. When the volume of tumors reached 100 mm^3^, the mice were randomly divided into two groups (7 mice in each group). Then, PBS or CP-EPS8-NLS were intraperitoneally injected into the mice every other day. **b** The volume of each tumor was measured every other day for 22 days. **c** Body weight of the mice. **d** The volume of each tumor was measured after tumor extraction from the mice at the experimental endpoint. **e** Image of tumors excised from mice after 22 days of treatment. **f** EPS8 and Ki67 in tumor tissues at the end of the experiments using IHC staining

The correction does not have any effect on the results or conclusions of the paper.
