# Supplementary figures and images for: A synthetic cell-penetrating peptide derived from nuclear localization signal of EPS8 exerts anticancer activity against acute myeloid leukemia
Source: J Exp Clin Cancer Res. 2018 Jan 22;37:12. doi: 10.1186/s13046-018-0682-x (PMC5778704; doi:10.1186/s13046-018-0682-x)

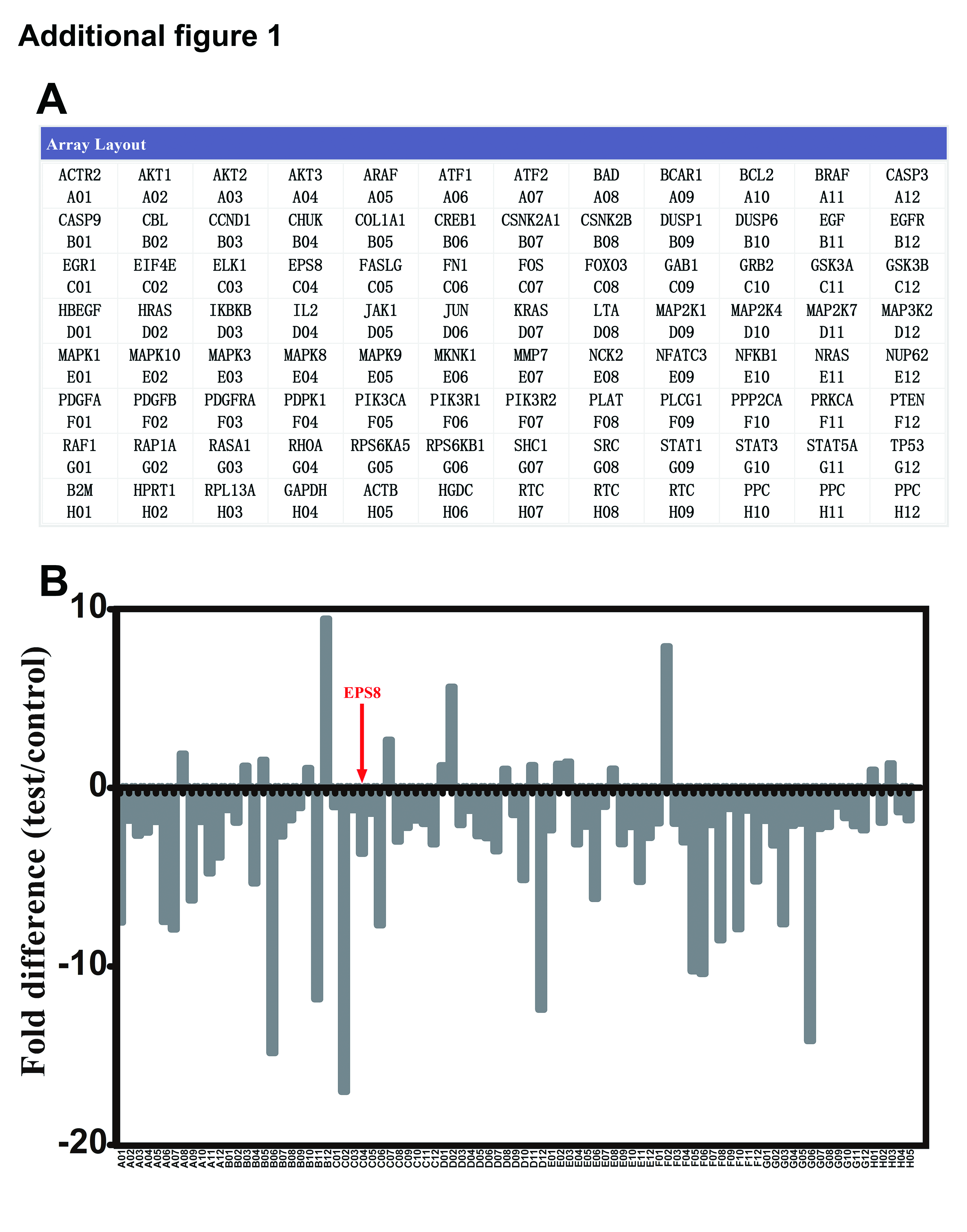

Supplement: Supplementary file 1 — Changes in EGF/PDGF signaling pathway targets analyzed with a RT2 profiler™ PCR assay. A Array layout of the RT2 profiler™ PCR assay. B Changes in EGF/PDGF signaling pathway targets in KG1α/sh1 cells compared with those in KG1α/NC cells. (TIFF 6966 kb) [file 13046_2018_682_MOESM1_ESM.tif]

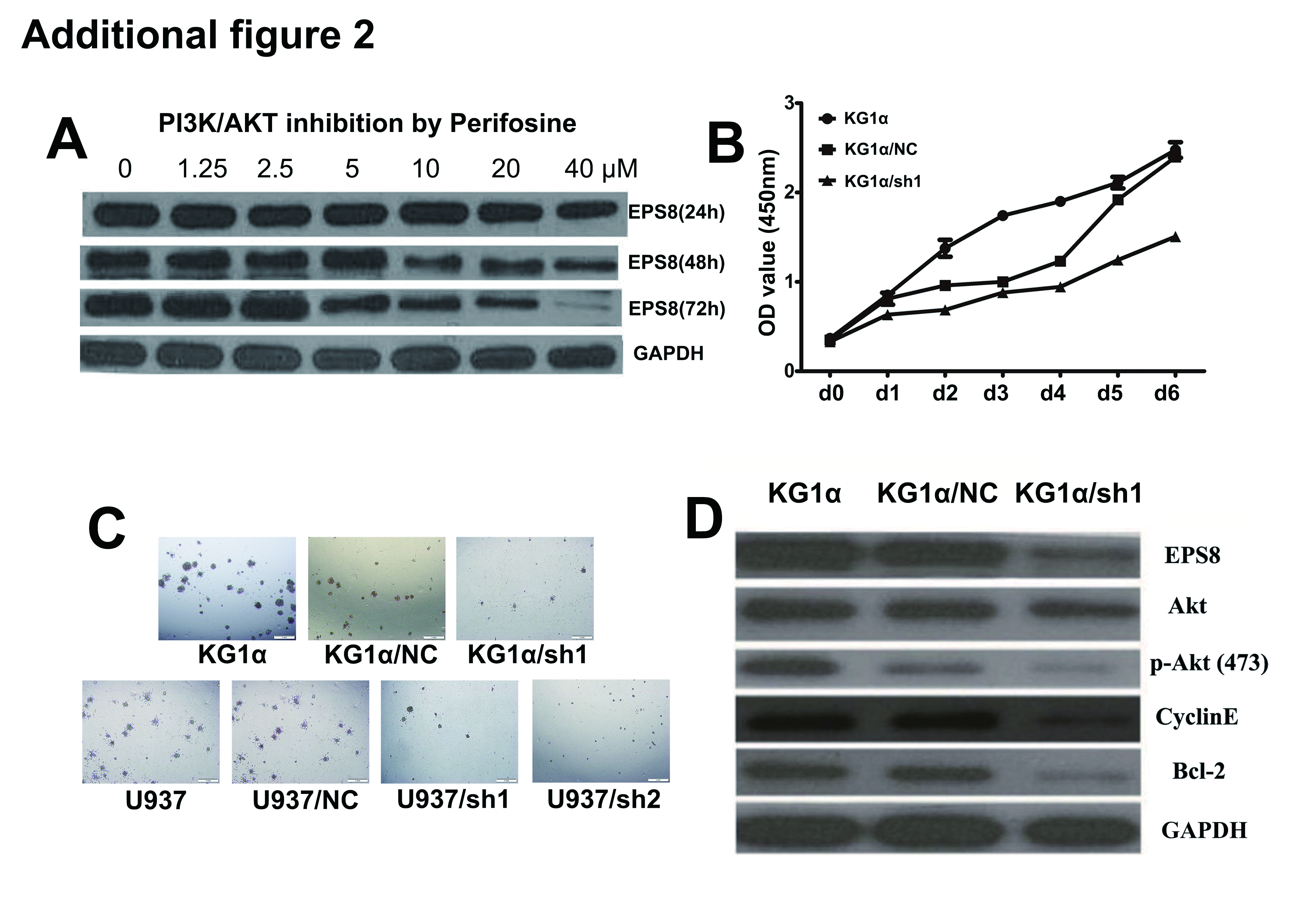

Supplement: Supplementary file 2 — The ability of EPS8 to influence AML cells survival. A Changes in EPS8 expression levels after treatment with increasing concentrations of the Akt inhibitor (perifosine) (0 to 40 μM) for 12 and 24 h analyzed by western blot. B Proliferation ability of KG1α cells after EPS8 knockdown. C Colony formation analysis in parental U937 cells or KG1α cells compared with shRNA1- and NC shRNA-infected cells. D Changes in EPS8 associated signaling pathways after EPS8 knockdown analyzed by western blot. (TIFF 7639 kb) [file 13046_2018_682_MOESM2_ESM.tif]

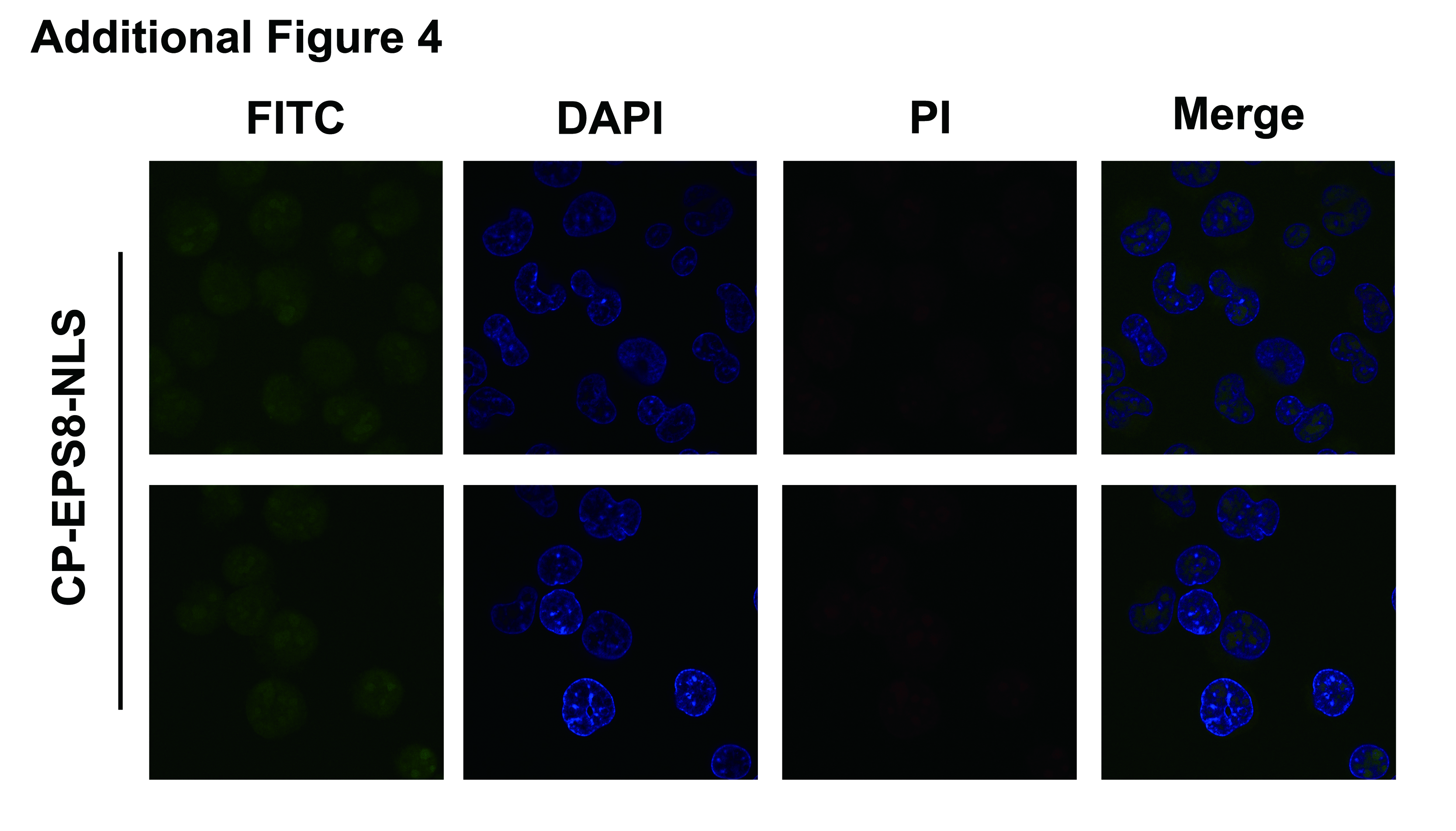

Supplement: Supplementary file 3 — U937 cells treated with CP-EPS8-NLS for 8 h and observed under a laser confocal scanning microscope. (TIFF 7912 kb) [file 13046_2018_682_MOESM3_ESM.tif]

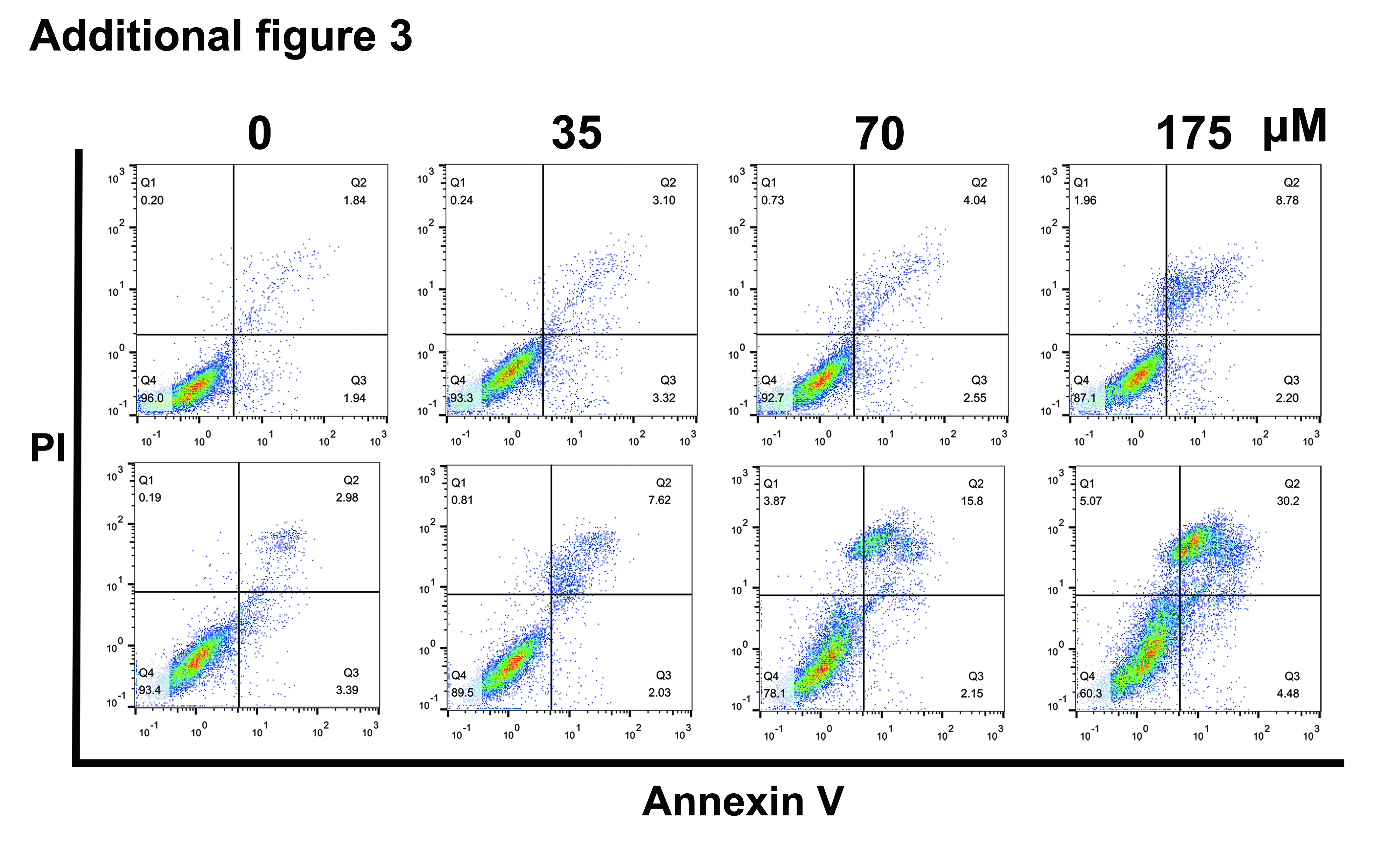

Supplement: Supplementary file 4 — Percentage of apoptotic KG1α cells after CP-EPS8-NLS treatment for 24 and 48 h. (TIFF 4126 kb) [file 13046_2018_682_MOESM4_ESM.tif]
